# Defining a Farmed Animal

**DOI:** 10.3390/ani16213520

**Published:** 2026-11-06

**Authors:** Katie Javanaud, Joseph Poore

**Affiliations:** 1 Independent Researcher, Manchester, UK; katiejavanaud@gmail.com; 2 Department of Biology, University of Oxford, Life & Mind Building, S. Parks Road, Oxford OX1 3EL, UK; 3 Oxford Martin School, University of Oxford, 34 Broad Street, Oxford OX1 3BL, UK

**Keywords:** farmed animal, farm animal, livestock, aquaculture, laboratory animal, traction animal, conceptual definition, agricultural statistics, legal definition, animal welfare

## Abstract

The categorisation of animals as ‘farmed’ determines whether they are included in statistical reporting and thereby the reported scale of animal agriculture. It also determines the degree of protection they are afforded under welfare legislation. However, the boundaries of this category have not been systematically examined. Definitions in law and practice vary across and within countries and are often based on arbitrary lists of taxonomic groups, intended uses, and production methods. Here, we critically explore the concept and identify the essential characteristics of a ‘farmed animal’ as one that: (1) is sustained and/or grown under human control; (2) is kept primarily to produce material goods; and (3) is kept primarily for profit, economic security, or sustenance. This definition supports consistency in a rapidly changing global farming system. However, we argue farmed status should not determine the level of welfare protection an animal receives: instead, welfare protections should reflect animals’ capacities for positive and negative experiences. Our definition provides legislators, researchers, and animal advocacy groups with a basis for revising and aligning their understanding of farmed animals, improving the visibility of excluded animals, and supporting collective action to improve their lives.

## Simple Summary

Humans are farming more animals in more ways than ever before. However, existing definitions of what counts as a ‘farmed animal’ are highly inconsistent, incomplete, and often unreflective. Resultantly, countless animals are missed from statistical databases intended to describe animal agriculture, from laws governing the treatment of farmed animals, and from public consciousness. Here we critically assess existing definitions and build a new definition of a ‘farmed animal’ based on essential features common to all. If this definition is adopted, it could support amendments to animal welfare legislation and drive revisions in statistical reporting worldwide, affecting societal understanding of the true extent of animal agriculture today.

## 1. Introduction

Most people can list animals which are typically farmed: chickens, turkeys, pigs, sheep, and cows. Similarly, most people can differentiate between farmed animals, companion animals, laboratory animals, and free-living animals. This might be taken to signify that the concept of a ‘farmed animal’ is unproblematic, requiring neither scrutiny nor refinement. However, we argue that there are moral and practical reasons to re-examine the concept and develop a more robust account of what it means for an animal to count as farmed.

Firstly, different countries and states use their own definitions of a ‘farmed animal’ as the basis to include or exclude animals from standards governing their treatment. In practice, the same species can receive entirely different welfare provisions depending on where they are located and based on that location’s definition of a ‘farmed animal’.

Secondly, the emergence of new global markets for animal products means that we are farming more species in more ways than we did historically [1]. Our definition of a farmed animal needs to keep pace with these emerging trends.

Thirdly, there is no consistent accounting of how many animals are farmed and current estimates report on different measures with different scopes. World Animal Protection estimates “more than 80 billion land-based animals are farmed every year” [2]. Our World in Data claims “more than 100 billion animals are killed for meat each year” [3]. And 80,000 Hours states “every year we kill somewhere between 400 billion and 3 trillion vertebrates” [4]. A clear conceptual definition of a ‘farmed animal’ would underpin a consistent re-estimation of the number of farmed animals, essential for those dependent on such data.

To our knowledge, no published study has developed and tested general criteria for determining membership of the category ‘farmed animal’ (see our systematic search for prior work in Appendix A). Harfeld [5] asks “What is a farm animal?”, but examines the mental, behavioural, and social characteristics of farmed animals, rather than developing criteria for membership of the category. White [6] notes that Australian jurisdictions do not share a common definition of ‘farmed animal’, but does not develop a consistent definition. Zeifert [7] uses ‘farm animal’ as an example of a category with fuzzy boundaries, but does not seek to clarify the boundaries. Pfau-Effinger & Sebastian [8] show that species can be re-categorised between ‘farm animal’ and ‘pet’ as their functions change.

Comparable definitional work has been undertaken in related areas. Décory [9], Lord et al. [10], Spengler et al. [11], and others, for example, propose and develop criteria-based definitions of domestication. Tian et al. [12] demonstrate the legal consequences of such criteria. What remains unresolved is the corresponding question for ‘farmed animals’: which animals belong within the category, on what basis, and whether a general set of criteria can define its boundaries.

With the aim of encouraging the adoption of a more reflective and conceptually robust account of the concept, here we:

1. Explore how farmed animals are conceived in legislation and by research organisations;
2. Propose an alternative definition;
3. Apply the proposed definition to a range of cases; and
4. Identify implications of adopting our proposed definition.

## 2. Current Conceptions of Farmed Animals

In this part, we examine usage of the term ‘farmed animal’ in legislation in the EU, U.S., and China (which are some of the largest producers of animal products globally) as well as by the Food and Agriculture Organization of the United Nations (FAO).

### 2.1. Farmed Animals as Understood in EU-Wide Legislation

Council Directive 98/58/EC [13] is foundational legislation for animal welfare in the EU. It establishes a general duty of care to farmed animals across all member states to prevent “unnecessary pain, suffering, or injury” [13].

The legislation uses a purpose-based conception of a ‘farmed animal’ as “any animal (including fish, reptiles, or amphibians) bred or kept for the production of food, wool, skin, or fur, or for other farming purposes”. This definition determines which animals are covered by the legislation. However, Article 1 of the Council Directive lays out four instances in which the provisions of this law do not apply:

1. to animals living in the wild;
2. to animals intended for use in competitions, shows, cultural, or sporting events or activities;
3. to experimental or laboratory animals; and
4. to any invertebrate animal.

Reasons for these exclusions are not supplied in the Directive. Exclusion 2 is problematic because of the scale on which pheasants, partridges, and other birds are bred for release and subsequent hunting in the EU in conditions akin to any other poultry farming [14,15]. Further, this exclusion appears inconsistent with other EU legislation: enterprises that rear gamebirds can qualify for farm subsidies under the EU’s Common Agricultural Policy [16]. Exclusion 3 is also problematic if it refers to the breeding of animals for sale to labs—again these animals are often raised in conditions akin to other farmed animals, albeit with potentially higher biosecurity. Exclusion 4, which excludes invertebrates, is also not justified in the Directive, given the very large numbers of decapod crustaceans now farmed [17], and despite scientific evidence supporting sentience in some invertebrate groups, notably cephalopod molluscs and decapod crustaceans [18,19].

Although the Directive begins with a broad, purpose-based conception of ‘farmed animals’, its subsequent exclusions progressively narrow the animals to which welfare protections apply. The result is that the operational meaning of a ‘farmed animal’ is substantially narrower than the Directive’s initial definition suggests.

### 2.2. Farmed Animals as Understood in U.S. Law

In the U.S., one of the principal federal statutes governing animal welfare is the Animal Welfare Act [20]. However, it largely excludes animals raised for agricultural purposes from its protections. Instead, farmed animal welfare law primarily operates at the state level. A second important federal statute is the Humane Methods of Slaughter Act, governing farmed animal treatment at slaughter, but this applies only to “cattle, calves, horses, mules, sheep, swine, and other livestock” [21], excluding poultry, fish, and crustaceans. Protection for these animals depends on other federal provisions and state legislation.

At the state level, definitions of farmed animals used in agricultural and animal-related legislation vary considerably. Some states place substantial weight on the *purpose* for which they are kept (e.g., the Tennessee Code on Agriculture and Horticulture states that “‘livestock’ means all equines as well as animals that are being raised *primarily* for use as food or fiber for human utilization or consumption including, but not limited to, cattle, sheep, swine, goats, and poultry.” [22]). Others identify farmed animals primarily through their species membership (e.g., Texas and Georgia) [23,24]. Others consider intended use in conjunction with species membership (e.g., Ohio). These definitions may also apply only within a particular statute or regulatory context rather than uniformly across a state’s law. The result is that the same species, kept for the same purpose, may be excluded or included based on which state it is produced in. There are many problematic implications of this [25] (e.g., consumers may expect uniform welfare provisions for animals of the same species or for animals performing the same function, but in fact this does not hold).

### 2.3. Farmed Animals as Understood in Chinese Legislation

The Animal Husbandry Law of the People’s Republic of China [26] is the main piece of legislation on the production of terrestrial animals, primarily focusing on food safety and disease-risk management, but also including some welfare provision. It uses a catalogue approach to identify farmed animals and currently lists 33 recognised kinds of “livestock and poultry”. The list includes species widely farmed globally (e.g., pigs, cattle, sheep, chickens, and ducks) as well as other species including reindeer, ostrich, and emu. Mink, silver fox, arctic fox, and racoon dogs are also listed for fur production. The law also gives partial coverage to bees and silkworms. Terrestrial species not on the list are then considered wild animals.

This classification was highly significant: in 2020, China banned the consumption of terrestrial wild animals. Terrestrial animals not classified as ‘farmed’, such as bamboo rats, civets, and porcupines, could no longer be farmed for consumption despite having established commercial farming industries. Within China therefore, the definition of a terrestrial farmed animal has been mandated by the state with substantial implications.

There are still edge cases in the categorisation. Dogs, of which around 10–20 million are killed for meat each year, are excluded, and appear to be considered as pets, justified by the government because cultural traditions around eating them are changing [27]. The catalogue also excludes reptiles and amphibians, some species of which are now classified as aquatic species and remain farmed in large numbers [28].

### 2.4. Farmed Animals as Understood by the FAO

Composed of 194 member states, the FAO shapes how agricultural censuses and surveys are conducted worldwide, collates data to produce global statistics, and drives research to improve food security. However, the FAO does not offer a general definition of a ‘farmed animal’. Instead, it uses the related concepts of ‘livestock’ and ‘aquaculture’.

The World Programme for the Census of Agriculture (WCA), which occurs every ten years, is the FAO’s main global statistical programme, substantially shaping what agricultural data are collected by countries. The programme’s definition of ‘livestock’ has changed substantially over time. In the 1930 to 1970 censuses a species list was used which gradually expanded over time [29,30,31,32]. The 1930 census, for example, included just horses, mules, asses, cattle, sheep, goats, swine, and bees [32]. The 1980 census introduced a definition of livestock as “all animals kept or reared mainly for agricultural purposes” [33]. The 2000 census removed the definition, reverting to a species list [34]. The 2010 census then reinstated a general definition as “all animals, birds and insects kept or reared in captivity mainly for agricultural purposes” [35], which holds in the current 2030 census although the species listed in the Annex has expanded over time to now include virtually all possible farmed species (even cats and dogs when “raised for food or other agricultural purposes” [36]). We also note that the FAO has shifted towards a *purpose* or functional definition, which demonstrates such criteria are feasible to implement in data collection at a global scale.

Ultimately, the census counts animals only if they can be attached to an agricultural holding. The census rule for attributing animals to holdings is of particular relevance here: “a holding is considered to be raising an animal if it has primary responsibility for looking after the animal on a long-term basis and for making day-to-day decisions about its use”. This is a test of *human control*, demonstrating a control-based criterion, too, is feasible to implement in data collection at a global scale.

The FAO defines ‘aquaculture’ as the farming of aquatic organisms where farming implies “some form of intervention in the rearing process to enhance production, such as regular stocking, feeding, protection from predators etc.” and where there is “individual or corporate ownership of the stock being cultivated” [37]. This definition is strong insofar as it emphasises both human intervention in the lives of farmed animals and their commodification. However, we note that this definition, if applied consistently, would include animals in zoos, aquaria, and other settings generally not considered as farming. Further, we note the FAO’s FishStat database does not reliably comply with its own definition: e.g., crocodilians which are farmed for their meat and skin are included in FAO’s wild capture production statistics [38].

### 2.5. Summary

In the cases assessed, the definition of a farmed animal is highly important for legislation and for agricultural statistics. However, the definitions can be arbitrary; poorly justified; inconsistent across countries, states, and legislative instruments within the same country; and inconsistently applied in practice. This demonstrates the need for a more systematic approach to understanding the concept.

We also found that purpose-based and human-control based definitions are already applied at the global scale. This indicates that these components of the definition are operationalisable in countries worldwide.

## 3. Materials and Methods

In this section we describe our methods for building a new definition of a ‘farmed animal’. We first considered both essentialist and non-essentialist approaches. An essentialist approach would use a set of criteria to create the definition, whereas, according to a non-essentialist approach, there is no single comprehensive set of criteria which all farmed animals satisfy, and context and the term’s usage also contribute to whether an animal should count as farmed. Whilst this open-endedness might seem reasonable in the context of rapidly changing farming practices in which new technologies might transform our current understanding of agricultural production [39], it also leaves the parameters of the concept unclear. If the goal is to chart a path between too narrow and too broad an account of what makes an animal farmed, then this can be achieved without jettisoning the idea that there is some determinate set of features held in common by every farmed animal. We contend that where the definitions of farmed animals examined in Section 2 are flawed, they have failed not because they are essentialist *per se* but rather because they have misidentified which features are the essential ones for an animal to count as farmed.

We also considered the possibility that an animal could be both classified as farmed and classified in another way (e.g., as a pet) at the same time. However, this would create challenges for reliably enumerating the number of animals farmed in statistical reporting and would also create challenges in deciding which legislation they should be included in. We therefore concluded that, at least for practical reasons, animals should only be defined as either farmed or not farmed at any point in time.

We identified general principles that our conceptual definition should satisfy, drawing on the results from Section 2 and prior frameworks for developing and refining concepts [40,41]. An effective definition should:

- Be sufficiently restricted so that distinctions—such as between farmed animals, companion animals, and animals used for research or entertainment—retain their meaning and function.
- Be parsimonious.
- Have clear conditions that can be applied unambiguously and consistently.
- Be independent of considerations that *do not* determine whether an animal is farmed (e.g., the consequences for welfare standards).
- Be practical to implement (e.g., in a typical agricultural survey).

To arrive at the definition, we started by identifying widely accepted cases of farmed animals, for which we used the animals listed in the FAO’s 2030 World Census of Agriculture (WCA) [36] and the ISSCAAP aquaculture taxonomy [42] (see Appendix B). We also considered the broader FAO approach to identifying livestock and aquaculture animals, which includes the purposes for which they are kept and the nature of human management or intervention involved (see Appendix B). We treated the FAO’s definition as widely accepted, as an international organisation of the United Nations, representing and engaging with all countries worldwide, but did not treat it as authoritative. This is because, although the main species and purposes within the FAO’s concept of a farmed animal have been relatively stable, new species, purposes, and definitions have been added and removed over time (Section 2.4). Put differently, we treated the FAO as containing the uncontested core of the concept but questioned cases that could be categorised differently. We then examined which characteristics were common to paradigmatic examples of farmed animals and which were not shared by animals belonging to other categories (e.g., animals kept for companionship, animals kept for entertainment, or animals kept to provide services to humans). This produced the three essential characteristics we list below, together with a wider set of additional features commonly shared by farmed animals, which we also list below.

We then assessed seven cases against this definition in depth and describe them below. The cases were identified through critical reflection on the FAO species and functions, as well as reflection on other classes of animals such as pets. We assessed five cases of animals considered farmed in other contexts but not generally included in FAO data: animals farmed for subsequent hunting, animals farmed for the pet trade, animals farmed for subsequent use in research, animals held captive for use in traditional/indigenous medicine, and animals ‘culled’ in the farming sector. We also assessed two cases of animals counted as farmed in FAO data but which our definition challenged: pollinator insects and animals used to provide traction or transport.

We then applied our definition to a wider range of cases, which we list in Table A1. These include challenging edge cases such as wild-caught fish and crustaceans kept alive, sometimes for weeks, until they are sold for consumption.

This approach helped us distinguish between characteristics that are essential to the definition and those that are commonly, but not invariably, associated with it. For example, farming need not occur at scale, nor need it involve domestication or human-directed genetic change, even though in practice it often does. Take bear bile farming, for example, which often involves bears captured from the wild and occurs on small scales [43].

## 4. A Proposed New Definition of a ‘Farmed Animal’

We propose that a ‘farmed animal’:

1. Is sustained and/or grown under human control; and
2. Is kept primarily to produce material goods; and
3. Is kept primarily for profit, economic security, or sustenance.

**Figure 1:**
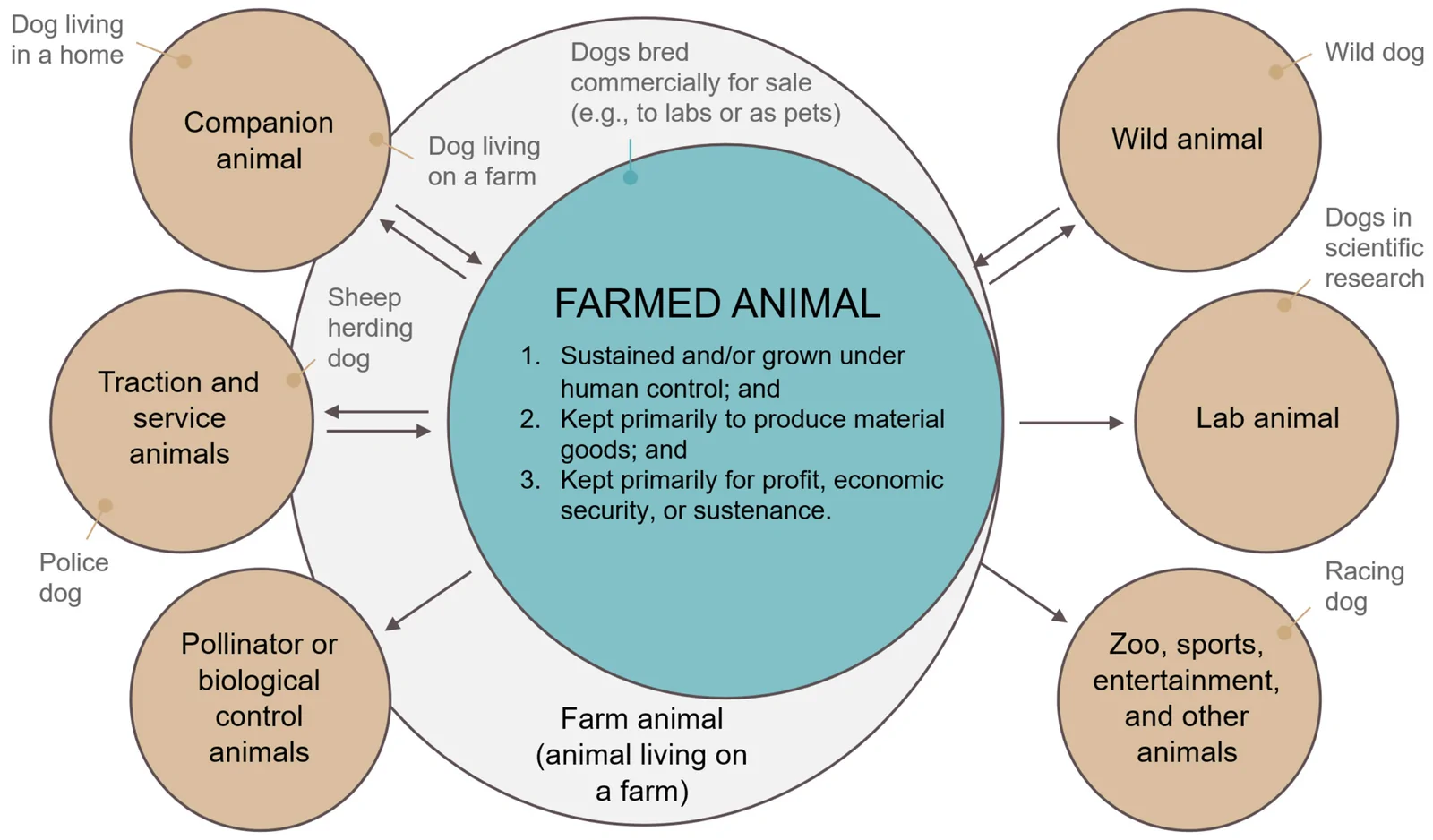
A conceptual overview of the concept of a ‘farmed animal’ and its boundaries. Arrows indicate that animals can move from being farmed to another category and/or vice versa (e.g., fish, captured from the wild and reared in ponds, would change categorisation from wild to farmed). Grey area indicates the related concept of a ‘farm animal’ (i.e., an animal living on a farm). We illustrate the figure with an example of dogs being classified in different ways.

We contend that all three must be satisfied for an animal to count as farmed. Classification is based on an animal’s current circumstances, and some animals may move between farmed and not farmed over their lifetime (e.g., juvenile fish caught wild then fed to maturity in farms) (Figure 1).

This definition meets our criteria of an effective definition, retaining widely accepted distinctions, being parsimonious, being independent of other considerations (e.g., around welfare provision), is clear to apply (see Table A1), and would be practical to implement (given similar human control and purpose-based definitions are already applied by the FAO at the global scale, see Section 2.4 above). The proposed definition aims to supply a framework which allows for the systematic, unambiguous, and comprehensive classification of farmed animals. We anticipate that the adoption of the proposed definition would invite ethical questions and could have both normative and regulatory consequences.

In the following sub-sections, we expand on the reasoning behind each criterion.

### 4.1. Sustained and/or Grown Under Human Control

In many cases, farmed animals spend the entirety of their lives under human control: from the moment they are conceived (sometimes through a process of artificial insemination) to the moment they are slaughtered. Their experiences and well-being are shaped both by direct human management and by legal frameworks governing matters such as cage sizes, stocking densities, handling, husbandry, and permitted slaughter methods.

Living under human control is a matter of degree. Whilst there are obvious instances of non-humans living under human control, some of the relationships between humans and non-humans are much harder to classify. Indeed, some ethicists have suggested that, particularly in the Anthropocene where human interference in non-human lives is somewhat inevitable, the distinction between captive and free-living animals is increasingly unclear [44,45]. Yet, whilst there is undoubtedly some truth in the claim that human activity has become so pervasive as to have some level of influence over almost all other life forms, we nevertheless see value in continuing to distinguish between animals who retain substantial autonomy and those whose lives are firmly under human control.

For our purposes, therefore, we take animals to be *living under human control* when:

- their physical location or range is substantially determined by humans who seek, in some way, to restrict or direct their movement; *and*
- when their opportunities for expressing natural behaviours are substantially determined by human decisions or interests; *and* when
- their welfare is largely determined by human behaviours, decisions, or standards.

Because there are many instances where animals are under human control but not necessarily farmed (e.g., a wild-caught fish who is alive for a few minutes on the deck of a fishing boat), it is necessary to stipulate some further conditions contributing to an animal’s status as ‘farmed’. We contend that, for an animal to be farmed, the animal must be *sustained and/or grown under human control*. We take *being sustained* to mean that the animal’s life is sustained through active measures taken on the part of humans. In other words, whilst flailing fish may be temporarily sustained until their oxygen levels become too depleted on-board deck, they would not count as farmed for the duration of time they are under human control even though they are kept to produce material goods and for profit, economic security, or sustenance because no active measures are taken to sustain the animals. On the contrary, they are only incidentally—and for a very temporary period—sustained.

In the case of wild-caught fish which are kept alive, sometimes for several weeks before slaughter to provide fresh meat, such animals would count as ‘farmed’ only if they became actively managed by humans who were feeding, treating, or breeding from them. Merely keeping them in an environment—water—in which their lives could be prolonged would not be enough to change their status from free-living to farmed but would rather count as an extended period of transportation of a wild-caught animal. If they do start being fed, fattened, or bred, they become farmed. Incidentally, this also aligns with the FAO’s approach and definition of “capture-based aquaculture” [46].

### 4.2. Is Kept Primarily to Produce Material Goods

A second essential feature all farmed animals share is that they are kept primarily to produce material goods. That the good produced should be *material* is important, as this qualification allows us to distinguish between farmed animals and those kept to produce non-material goods or services. For example, we would not classify animals kept solely for entertainment or for the sake of scientific research as farmed animals, even though they are clearly kept to produce some good (e.g., entertainment, stimulation, or the advancement of scientific knowledge).

What counts as a material good? Material goods may be derived from an animal—such as milk, eggs, honey, or silk—or may consist of part or all of the animal’s physical body. Importantly, an animal may itself constitute the material good being produced or supplied, whether alive or dead. So, for example, rodents may be farmed prior to being used in scientific research, but cease to be farmed once they are kept primarily for research. Again, dogs may be farmed (where the material good produced is their own physical body) prior to becoming companion animals. These examples also illustrate how animals may count as farmed for a temporary period.

Whether production is ultimately successful or not should not change their classification as farmed. Animals who die or are culled before producing an intended good remain farmed under this criterion. Chicks are farmed until their sex is known, at which point males are generally killed as they are no longer profitable.

Animals used solely to provide labour, including traction animals not primarily farmed for meat or milk, are not classified as farmed under this definition: the crops or other goods produced through their labour are not material goods produced from, or constituted by, the animals themselves. In cases where the manure from animals kept primarily for traction is used (e.g., for fertilising soil), the animal should not be classified as farmed since they are not kept primarily to produce the material good.

We note that in the World Census of Agriculture, enumerators already use such a test, asking what the “main reason for the animals being kept” is [36]. Where the keeper’s own account is unclear, enumerators check against proxies such as the share of income that each purpose supplies (we discuss the use of proxies further in Section 5.8).

### 4.3. Kept Primarily for Profit, Economic Security, or Sustenance

Finally, we hold that all farmed animals are kept primarily for profit, economic security, or sustenance. Economic security includes keeping animals as savings, insurance, exchangeable assets, or buffers against future material need [47]. Sustenance includes the provision of basic material goods used to maintain life, health, or material subsistence, including food, clothing, fuel, and comparable necessities, whether supplied to the keeper, the keeper’s household, or to other people.

By identifying profit, economic security, and sustenance as the primary reasons for which animals are kept, we count even animals produced on small scales as farmed. Profit need not be the sole motivation: farmers may also be motivated by family tradition, the desire to provide food to their communities, or a preference for rural life [48]. What matters is that the animal is kept *primarily* for profit, economic security, or sustenance, rather than that these are the keeper’s only motivations. An animal may therefore generate income without being kept primarily for profit, as where a companion dog is bred from once but remains primarily kept for companionship. In cases where it is not possible to discern the primary motivation for keeping an animal—either by directly asking the keeper or through the use of sensible proxies—or in cases where there may be multiple equally strong reasons for keeping animals (amongst them profit, economic security, or sustenance), how should the animal be classified? Here, it might seem that the classification is somewhat arbitrary but we contend that this arbitrariness can be avoided by invoking normative considerations of the implications of a classification one way or the other. In other words, in the rare—but technically possible—case of it being impossible to establish a primary reason for which the animal is kept, the classification should be based on practical considerations (e.g., welfare implications). We note, however, that such practical considerations are compelling only to the degree that welfare provisions or standards are linked to classificatory boundaries and that, in fact, the risk of arbitrariness here should only serve to reinforce our argument that it is more rational to link welfare standards to sentience, and the capacity for positive and negative welfare states.

### 4.4. Additional Non-Essential Features Commonly Shared by Farmed Animals

Whilst being sustained and/or grown under human control, being kept primarily to produce material goods, and being kept primarily for profit, economic security, or sustenance are the three essential features of farmed animals, there are a number of additional (non-essential) frequently shared features. These include, but are not limited to, animals:

- having their genes shaped through domestication and selective breeding, sometimes to such an extent that they cannot live independently of humans;
- being produced in large numbers or at high levels of intensity;
- being rendered largely invisible by the system in which they are kept, as happens when farmed animals are kept out of public view in industrial facilities;
- having their individuality overlooked or systematically obscured (as happens when aquatic species are quantified in tonnes of liveweight rather than as individuals, or as happens when animals are identified through numbered tags and not by names or personal characteristics); and
- having their longevity determined by their productive value (as when hens are slaughtered once their egg-laying capacity becomes unprofitable, or when milk producing cows are retained for longer than their meat producing counterparts).

## 5. Applying Our Definition to Unconventional Cases

In this part, we apply our definition to a range of cases of animals all living under human control. In Table A1 we report a broader list of cases and provide concise justifications for whether animals in these contexts should be classified as farmed or not.

### 5.1. Animals Raised to Be Hunted

Around the world, many undomesticated species are bred and raised in captivity to be subsequently hunted [1]. According to the South African government, there are at least 8000 captive lions in South Africa [49]. Other African species raised for captive-hunting include: the southern white rhinoceros, wildebeest, impala, springbok, oryx, and Barbary sheep. Still other species, including some which are not native to any part of the African continent such as tigers, fallow deer, and Eurasian wild boar, are bred and raised either in caged environments or in restricted and predator-controlled environments for the purpose of trophy hunting and bloodsport experiences [1].

While these species are omitted from databases focused on agriculture and domesticated species, such as the Food and Agriculture Organization Statistical Database (FAOSTAT), failure to count them amongst farmed animals is an oversight for organisations seeking to deliver comprehensive insight and knowledge of the agricultural sector as a whole. This is especially so given that captive-hunting often produces meat, hides, furs, and feathers.

Taking the case of captive-bred lion hunting, for example, it is clear that all three essential criteria proposed in our definition of a farmed animal are met:

- they are both *sustained and grown under human control*—including after they are released into designated hunting camps, where their movements and conditions remain monitored and managed [50];
- they are *kept primarily to produce a material good*—namely their physical bodies, as trophies;
- they are *kept primarily for profit*—with each shoot marketed in the region of $20,000 to $40,000 [50,51].

However, a wide range of animals are bred specifically for bloodsport purposes around the world, not all of them living their entire lives under human control. For example, in Europe, the practice of raising gamebirds in captivity before releasing them into wild, open spaces and then shooting them is long-established. In the UK alone, approximately 40 million birds are released annually ahead of the shooting season [52]. Because we have argued that an animal’s status as farmed need not be permanent, if animals are captive-bred for the purpose of hunting and then released, they should also count as farmed for the period *prior to their release*, provided that all three essential criteria are met. Of pheasants that are shot, the ~6–7-week captive-rearing period represents roughly a quarter to a third of their total life [53]. We note that once released, the course of their lives continues to be heavily influenced by humans who offer supplementary feeds, deploy extensive predator and parasite control measures, and ultimately shoot them [54]. Nevertheless, the three criteria for living under human control are not substantially met during this time, and these animals are therefore not farmed after they are released.

### 5.2. Animals Raised and Sold for Companionship

Companion animals are not farmed animals. Take the quintessential example of a companion animal, a dog: although they are sustained under human control, they are not kept to produce material goods, nor are they kept for profit, economic security, or sustenance. If anything, they are kept for immaterial goods (i.e., for the positive relationships we can share with them), but quite often they are kept for their own sake, in recognition of their own intrinsic value.

However, where animals are bred specifically for sale as companion animals, then during this period they meet all three essential characteristics of farmed animals. In other words, animals can transition from being farmed animals to being companion animals (Figure 1). Similarly, animals can transition from being companion animals to farmed animals if the purpose for which they are kept shifts to one of production and profit. This might happen, for example, when a cat kept for companionship is used to breed kittens and starts being kept primarily as a source of economic security. This is different from scenarios in which a companion animal happens to procreate and the offspring are—incidentally—monetised.

It is noteworthy that, despite not being recognised as farmed in the legislative contexts examined in Section 2, animals bred, raised, and sold for companionship are increasingly understood as farmed in public consciousness. Indeed, the term ‘puppy farming’ is now widely used to refer to the (sometimes intensive) commercialisation of companion animals [55]. While not a core characteristic of a farmed animal, the scale on which companion animals are produced also lends weight to the idea that animals raised for companionship should be classified as farmed: in the USA alone, an estimated 2.6 million such puppies are sold each year [56].

### 5.3. Animals Destined for Use in Research

Animals bred or grown for sale to research labs are farmed animals, meeting all three criteria. However, their status changes to not farmed when they are in laboratories. This is because, although being grown and sustained under human control, these animals are not (typically) themselves productive of a material good. Rather, animals kept in research facilities are typically kept as a *means* of producing a material good (i.e., a drug) but do not themselves produce the physical good. They are instead primarily kept to produce an immaterial good (i.e., the advancement of knowledge and understanding). If animals do directly produce a material medicinal good, they would be classified as farmed: for example, bears farmed for bile (see section below). Animals bred “in-house” by research facilities are not farmed, as they are never sold or supplied as a material good.

### 5.4. Animals Held Captive for Use in Traditional/Indigenous Medicine

A wide variety of animal species are used in traditional/indigenous medicines. Where species are bred for multiple purposes, such as for both meat and medicinal uses, they may already be represented in FAOSTAT data. A good example of this would be the estimated four to six million donkeys slaughtered each year to supply the global trade in donkey skins [57], from which ejiao (donkey-hide gelatin), a traditional Chinese medicine believed to have medicinal properties, is produced, with the meat typically a secondary product [58].

However, species without a clear food use are often missed. The farming of bears for their bile, for example, clearly satisfies the three criteria we have proposed for counting as farmed. Not only are these animals *sustained under human control* but, notoriously, they are typically kept under extreme control in small cages where their most basic needs and instincts cannot adequately be satisfied [59,60]. They are *kept to produce a material good*—namely their bile—and are *primarily kept for profit*. The farming of bears for bile is now widely recognised as incompatible with their welfare and, thanks in part to sustained campaigning from organisations such as Animals Asia, bear bile farming is being phased out in some parts of Asia, such as in South Korea and Vietnam. Nevertheless, bear bile production remains a legal and active industry in large parts of Asia, particularly in China where production is concentrated.

Still other animals are farmed specifically for the traditional medicine market, such as scorpions and centipedes. We contend that until all farmed animals are counted, it will be impossible to obtain a clear picture of the extent of human agricultural practices and of our direct impacts on non-human animals.

### 5.5. Animals ‘Culled’ in the Farming Sector

FAOSTAT data provides a snapshot of the number of animals farmed (“stocks”) at a specific point in time. It also provides information on the number of animals slaughtered for meat annually. The ~7 billion day-old male chicks ‘culled’ annually around the globe who, on account of their sex, cannot produce eggs and, who, on account of their breeding, are judged unsuitable for meat production [61], are almost certainly excluded from annual stock numbers, and definitely excluded from slaughtered counts.

Male chicks should certainly be counted amongst farmed animals. However short their lives may be, they are sustained under human control from the time the eggs are laid. Until their sex is known, male chicks are kept to produce a material good, and for profit. It is only once their sex is determined that it becomes clear they will not be profitable, at which point they are culled. To exclude male chicks from the data on farmed animals therefore leads to a distortion of the facts surrounding the number of animals farmed.

### 5.6. Farmed Insects and Pollinators

FAOSTAT reports data on two types of insect: bees (as number of hives) and silkworms (as mass of cocoon). Neither are reported as number of individuals. FAOSTAT does not directly provide data on other farmed insects used either directly for human consumption or as part of animal-feed production (although they may appear in a “miscellaneous” category in the Food Balance Sheets). Cochineal scale insects (*Dactylopius* spp.)—which have various food uses—are absent from FAOSTAT and yet are likely farmed in the billions [62,63].

However we may feel towards insects, whether we judge them to have any moral standing or not, they are being farmed in vast numbers: they are grown and sustained under human control, are kept to produce material goods, and are generally kept for profit. It is therefore important that they should be recognised as farmed in law, policy, and social discourse even as a precursor to subsequent discussions about whether and to what extent any welfare provisions should be afforded to them.

It may transpire that the outcome of acknowledging just how many insects are farmed is moral indifference. Yet the aim of revising our concept of what constitutes a ‘farmed animal’ is not automatically and unquestioningly to elevate moral status or welfare standards but rather to recognise the true scale of animal agricultural production and, from there, build a platform for further ethical discussion.

We note, however, that FAOSTAT includes bees kept for pollination. This provides a rare case in which our definition excludes animals from FAOSTAT and illustrates why FAOSTAT serves only as a starting point. Bees kept for honey or other material products are farmed, whereas bees kept solely for pollination are not: they provide a service that enables crops to be produced, rather than to produce material goods derived from, or constituted by, the bees themselves. This does not prevent those same bees from having been farmed at an earlier stage while they were being commercially bred, reared, or supplied as living animals.

### 5.7. Animals Used to Provide Traction and Transport

Millions of animals are used for traction and transport [64]. The FAO’s World Census of Agriculture (WCA) includes these animals as farmed if they live on agricultural holdings and meet the purpose of providing draught. However, the WCA would exclude sheep herding dogs or cats used to control rodents. This highlights an inconsistency: traction animals, ratter cats, and sheep dogs are not classified in the same way yet share the same essential characteristics: they are under human control, are primarily kept for profit, yet none are primarily producing a material good—instead they are providing a service. In fact, if dogs and cats are farmed for meat (a material good), the WCA explicitly counts them as farmed animals, showing that purpose (service vs. material good) is a key distinguishing characteristic in global agricultural statistics.

Here, we argue it is more consistent to categorise animals primarily providing traction and transport as service animals and not as farmed animals. The implication of dropping the characteristic of being kept to produce material goods from our definition of farmed animals would be serious, leading ultimately to the collapse of numerous helpful ways of classifying animals. For example, animals kept for entertainment, for the provision of services, and animals kept in laboratories for scientific research are—like farmed animals—grown and sustained under human control and kept for profit or economic security. Hence, one way of retaining meaningful distinctions between our uses of animals is to include being kept to produce material goods in our definition of farmed animals. With traction animals, this leads to results that some people might find counterintuitive, but which are in fact more defensible and consistent.

### 5.8. Why It May Not Be Possible to Identify Every Farmed Animal

In Table A1, we attempt to provide an extensive list of cases in which animals are farmed. However, it is not comprehensive. The vastness of human use of animals, the many different contexts in which humans control, instrumentalise, and profit from animals, and the fact that technological innovations are re-shaping farming practices and animal husbandry all prevent us from writing a closed list of farmed animals. Our purpose here has been to provide a generalisable definition against which we can test whether an animal is farmed.

We acknowledge that there are instances in which it is not immediately obvious, or in which our intuition is divided over, whether an animal should count as farmed. This is partly because our definition presupposes the accessibility of human motivations, and while these are often clear, sometimes they are not reliably inferred. Indeed, whether an animal is kept primarily for profit, economic security, or sustenance may not be clear in some cases. How, for example, should backyard hens be classified? Whilst they may provide sustenance, even the animal’s keeper may be uncertain of their motives—keeping the animal for a mixture of reasons including companionship, sustenance, a desire for self-sufficiency, or the maintenance of rural traditions etc. To address this challenge, we suggest the use of sensible proxies to gauge motives where necessary (e.g., flock sizes to differentiate companion animals from farmed animals, or the revenue derived from the activities associated with the keeping of the animal).

Importantly, such proxies should be reflective of the economic differences between countries to capture the economic or subsistence implications of keeping even very small numbers of animals in poorer regions with more restricted access to income or nutrition. A similar approach is in fact already applied in the WCA guidelines, which is then adopted by each country: for example, in France, the threshold is 100 poultry, in Greece it is 50 or more, whereas in Ghana there is no lower limit and households with poultry are included [65]. Following the FAO, we contend that the use of context-dependent proxies, in conjunction with self-reporting as to the primary purpose for which animals are kept, are sufficient means of deciding whether to classify animals as farmed. The inclusion of context-specific proxies (i.e., classifying animals as farmed or not depending on different flock sizes in different countries) is important as a means of ensuring that our proposed definition is applicable in a variety of legal and economic contexts. It is important to note that such context-dependency need not introduce ambiguity over whether an animal should be counted as farmed. This is because the definition of a farmed animal can be consistently applied across different legal frameworks, whilst simultaneously allowing that different countries will have different thresholds against which to measure if an animal is kept primarily for profit, economic security, or sustenance.

We contend that even in vastly different cultural contexts our proposed definition remains pertinent: even in communities where reciprocity, co-operation, and the sharing of material resources fill the place of concepts such as profit or economic security, the importance of sustenance, and the need for material goods is universally acknowledged.

The proposed definition allows us to identify farmed animals using rationally defended criteria, rather than relying on more arbitrary methods (such as species membership) which have been shown to exclude, without justification, animals which are clearly farmed.

## 6. Implications

### 6.1. Implications for Legislative Bodies

The adoption of our proposed definition of a ‘farmed animal’ would necessitate reviews of, and in some cases, revisions to existing laws. The main piece of EU-wide legislation on farmed animals, for example, would need amending to include animals bred for release into the wild or captured from the wild into farming systems, animals farmed for sporting events and other similar activities, animals farmed for supply to labs, as well as farmed invertebrates. Similar changes would be required in the U.S. and Chinese legislation we investigated as well as in other geographies.

A worry is that by re-classifying animals as ‘farmed’, legal protections for some animals could be weakened, particularly given how much policy rests on this classification (Section 2). For example, pet animals may have stronger legal protections in some countries than farmed animals and while our definition would keep pets in homes as pets, it would re-classify pets being bred for profitable sale as farmed. If laws dictate that better veterinary care and more opportunities for exercise, socialisation, the expression of maternal instincts, etc., must be afforded to animals raised for companionship than to animals raised for slaughter, then it would be backward to insist they should be classified as farmed. Equally, lab animals have some of the highest biosecurity and veterinary requirements of all animals under human control [66,67,68], and while our definition would not affect the classification of animals in labs, it would re-classify animals bred for sale to labs as farmed.

Precedent from existing welfare reform, however, suggests that including more animals within the scope of welfare legislation has not weakened protections. In England and Wales, the Animal Welfare Act 2006 extended a proactive duty of care from farmed animals to other animals under human control; it did not weaken protections for farmed animals, and it allowed for different species to have specific provisions [69]. In the US, the federal Animal Welfare Act, originally limited to animals supplied to and used in research, was extended in 1970 and 1976 to animals used in exhibitions, sold at auction, or transported commercially, without any reduction in the protections already afforded to research animals [70]. There are other equivalent cases we are aware of, including in the EU [71], Norway [72], and Switzerland [73]. Despite this precedent, there is no guarantee future reforms which expand the number of animals and uses would not see weakening of welfare provision, and we explicitly acknowledge this as a potential risk. The EU explicitly guarded against reducing welfare provision by only allowing Member States to maintain the same or higher national standards when it extended its legislation to include more species [71]; while such safeguards may be important, they may also lead to over-protection of taxa like insects which—while farmed—are unlikely to merit the same welfare provision as other species.

We would, instead, argue that welfare provision should not be determined by how animals are classified. What counts as morally acceptable treatment of animals living under human control should be determined by their capacities for positive and negative welfare [74]. That is, the degree of legal protection an animal receives should principally be decided by the question of sentience [75]. This is increasingly reflected in legal reform: the EU extended its protections for animals used in science to cephalopods on the basis of scientific evidence of their capacity to experience pain [71] and the UK extended the scope of the Animal Welfare (Sentience) Act 2022 [76] to decapod crustaceans and cephalopods following a review of the evidence of their sentience [18]. Therefore, while the classification of animals matters now for welfare, there are good reasons not to base welfare provision on this, and evidence this is already changing. In this future, how animals are classified will remain important to policymakers, particularly for agricultural statistics, agricultural policy, human nutrition, and food security.

### 6.2. Implications for Research Organisations

The global adoption of the proposed definition of farmed animals would mean that countries would need to expand the species of animal covered in their agricultural censuses and surveys. Research organisations would then need to report the numbers of animals farmed, meaning that databases like FAOSTAT would be much more comprehensive and reflective of the full scale and scope of animal agricultural practices. Admittedly this would entail additional effort on the part of research organisations but would be justified by the benefits—not just for the countless farmed animals currently overlooked in policy, law, and social discourse, but also for the wider environment, and for human health, which are both deeply affected by animal agriculture.

Ultimately, the adoption of our proposed definition of farmed animals by leading research organisations would open the gateway to more accurate reporting of the data on farming practices. Access to this knowledge and data is essential to solving the multifarious problems associated with the farming of animals.

### 6.3. Implications for Consumers

Adopting a more accurate and reasoned definition of a farmed animal would bring to light the true number of animals behind the goods that are consumed. This number would include directly consumed animals as well as mortalities on farms, culled animals, animals farmed to be fed to other animals, and animals not counted in typical statistics. If effectively communicated (such as via product labelling, or through governmental or non-governmental campaigns), this information would allow consumers to grasp the full scale on which animal lives are affected by their choices for the first time. Individuals would see the number of farmed animals required, not only to produce goods directly for human consumption, but also the vast numbers behind products which are not directly for human consumption.

Beyond providing a more complete picture of our animal use, such transparency has ethical implications. By making visible animals currently excluded from agricultural statistics, the adoption of the proposed definition in law, policy, or social discourse could help stimulate further dialogues and debates about the legitimacy (or otherwise) of our use of farmed animals, the scale of their production, their treatment, and how our choices affect their lives. To the extent that reason and understanding inform ethical decisions, any such discussions may ultimately have implications for consumers who may either steer, or be steered by, revisions to farming practices or improved animal welfare standards.

Lastly, whilst it will be for empirical investigations to discover whether access to fuller information about the scale and nature of animal farming changes consumer behaviour, we contend that, regardless, such transparency is valuable in its own right, as consumers have a legitimate interest in understanding the systems that produce their food and other material goods.

### 6.4. Implications for Animal Advocacy Groups

Animal advocacy groups would be in a stronger position to communicate the full scale of animal farming and, rather than using caveated claims of the sort made by World Animal Protection, 80,000 Hours, and Our World in Data discussed earlier, they could simply state the total number of animals farmed, by species, and geography, using shared facts based on a consistent definition. This knowledge would be useful for animal advocacy groups in the design and delivery of their campaign work—allowing them to target harmful, entrenched, and extensive parts of the animal agricultural sector or else to target parts which are already showing vulnerability in the face of revised social attitudes (e.g., farming for fur/skins, farming for traditional medicines, or farming for bloodsports).

At the same time, knowledge of the full scale of animal farming would force the animal advocacy movement to confront difficult ethical issues surrounding the farming of particular groups of animals—notably those whose sentience is either unclear or unlikely, and those judged to be sentient and yet whose capacity for welfare (either good or bad) is thought to be very limited.

## 7. Conclusions

Today, our current best evidence shows that global farmed animal populations are rising rapidly. FAOSTAT reports global stocks of 35 billion farmed vertebrates in 2024, a 5-fold increase since 1961 [77]. As we have discussed, this likely underestimates the true number of animals farmed. These huge numbers, and this rapid change in the global system, underline the vital need for more critical reflection on what it means to be farmed. This will have implications for policies which use definitions of farmed animals, research organisations which enumerate and research farmed animals, consumers whose choices shape demand for farmed animals, and animal advocacy groups seeking to change the system.

Our work is the result of a conceptual analysis that has been tested with a large but not exhaustive number of cases. We encourage future work to further test it against new cases, particularly those that emerge as our relationship to farmed animals changes in the future, and for more people working in different contexts to apply and critically assess the criteria. In particular, a formal process assessing inter-rater agreement could be an important next step to support more widespread adoption of the definition, supporting greater harmonisation across organisations working in this area. We also encourage future work to re-estimate the number of animals farmed using our definition, to make the case for better and more harmonised statistical reporting, to critically assess global welfare standards and other legal protections given this framing, and to develop a broader categorisation of all animals.

We also hope our work invites greater critical reflection on how we categorise animals—as farmed, laboratory, companion, zoo, or free-living etc. Ultimately, whilst these categories and definitions are extremely important determinants of how we treat animals in different contexts, it is worth remembering that these classifications are mere human constructs, typically more reflective of our needs and interests than of the animals whose lives they so profoundly shape.

## Data Availability

The original contributions presented in this study are included in the article. Further inquiries can be directed to the corresponding author.

## Appendix A

To identify prior studies which defined or critically assessed the concept of a ‘farmed animal’ we followed the PRISMA framework for a systematic review. We used the following search string in Web of Science on 23 September 2026, which returned 782 studies:

TS = ((“farm* animal*” OR livestock) NEAR/3 (categor* OR classif* OR defin*)) OR TS = ((“farm* animal*” OR livestock) AND (pet OR pets OR “companion animal*” OR “laboratory animal*” OR “lab animal*” OR “research animal*” OR “working animal*” OR “draught animal*” OR “draft animal*” OR “service animal*” OR “wild animal*”) AND categor*)

We additionally searched for industry, governmental, or intergovernmental reports and other grey literature using the following Google search on the same date stopping after 20 pages of results, which led to 181 additional documents after removing duplicates within the search results and with Web of Science. We limited the search to pdfs to focus on publications and reports rather than websites:

(“farm animal” OR “farmed animal” OR livestock) (definition OR category OR classification) (pet OR “companion animal” OR “laboratory animal” OR “working animal” OR “wild animal”) filetype: pdf

For academic articles we screened abstracts, and for reports we screened summaries and opening pages. We included studies for full-text screening if they discussed the concept itself (definitions, criteria, membership, or differences between categories). We excluded studies that took categories as given (e.g., articles primarily focused on other topics like disease or environmental impacts) and excluded policy documents defining the category for policy reasons (which we discuss separately in Section 2).

Of the 963 records (782 from Web of Science and 181 from Google), 943 were excluded at screening, leaving 20 for full-text assessment. Of these, ten discussed conceptual problems with the category of a ‘farmed animal’ and its boundaries, but did not propose criteria for a new definition [5,8,78,79,80,81,82,83,84,85]. Five were surveys or experiments showing that individuals agree on the classification of typical farmed animals, but for species such as horses classification is more contested and driven by factors like utility [86,87,88,89,90]. The remaining five defined adjacent categories (wild animals, re-wilded animals, companion animals, and domesticated animals) [91,92,93,94,95].

## Appendix B

Here we list species and agricultural purposes of animals which are considered farmed according to the FAO. This was the departure point for our definitional work. Specifically, the terrestrial species were drawn from Annex 8 of the FAO’s 2030 World Census of Agriculture (WCA) [36]:

- Cattle, buffalos, and other bovine animals
- Sheep and goats
- Swine/pigs
- Horses, asses, mules, and hinnies
- Dromedary, Bactrian camel, Llamas and alpacas, other camelids
- Chickens, turkeys, geese, ducks, guinea fowls
- Deer, elk, reindeer and other ruminants
- Rabbits and hares
- Dogs and cats
- Ostriches and emus
- Other birds (e.g., pigeons)
- Other animals (e.g., primates, elephants, reptiles, zebras)
- Bees
- Silkworms
- Other insects and worms

The aquatic species were drawn from the ISSCAAP aquaculture taxonomy [42], limited to those for which FishStat records aquaculture production in 2024 [96]:

- Freshwater fishes (carps, barbels, other cyprinids, tilapias, other cichlids, miscellaneous freshwater fishes)
- Diadromous fishes (salmons, trouts, smelts, river eels, sturgeons, paddlefishes, shads, miscellaneous diadromous fishes)
- Marine fishes (flounders, cods, hakes, haddocks, halibuts, soles, miscellaneous)
- Crustaceans (shrimps, prawns, freshwater crustaceans, crabs, sea-spiders, lobsters, spiny-rock lobsters, miscellaneous)
- Molluscs (oysters, clams, cockles, arkshells, scallops, pectens, mussels, abalones, winkles, conchs, freshwater molluscs, miscellaneous marine molluscs)
- Miscellaneous aquatic animals (turtles, frogs and other amphibians, sea-urchins and other echinoderms, sea-squirts and other tunicates, miscellaneous aquatic invertebrates)
- Miscellaneous aquatic animal products (pearls, mother-of-pearl, shells)

As discussed in Section 2.4, we also included the “ranched” category from FishStat which includes tunas, bonitos, billfishes, and crocodilians.

The starting list of agricultural purposes drawn from the 2030 WCA [36] Annex 4, FAOSTAT production accounts [77], and FishStat [96]:

- Meat and offal
- Milk
- Eggs
- Wool
- Silk
- Hides
- Beeswax
- Honey
- Pearls
- Shells
- Draught
- Breeding

We note that we do not unquestioningly accept these species and/or functions as belonging to the category of a ‘farmed animal’ but rather view the list above as containing the core of the category and interrogate cases which could be categorised differently (e.g., we argue above that draught animals are better thought of as service animals).

## Appendix C

**Table A1:** Application of the proposed definition of a ‘farmed animal’ to various cases.

Case	Sustained and/or Grown Under Human Control	Kept to Produce Material Goods	Profit, Economic Security, or Sustenance	Category	Justification
Animals kept under human control to produce meat, milk, eggs, wool, hides, fibre, or honey for profit	Yes	Yes	Yes	Farmed	Paradigm case: the animal, body, offspring, or material product is the output
Backyard hens or goats kept primarily to supply the household or community with eggs or milk	Yes	Yes	Yes	Farmed	Commercial scale and sale are unnecessary where sustenance is the dominant purpose
Hens kept primarily as companions, with eggs eaten or given away	Yes	Yes	Not primary	Not farmed	Producing a material good is not enough when profit, economic security or sustenance is not a primary reason for keeping the animals
Animals kept primarily as savings, insurance, exchangeable assets, or a buffer against future material need	Yes	Yes	Yes	Farmed	Economic security captures animals functioning as stores of value even where no immediate sale or consumption is planned
Animals who die, are culled, or prove unproductive before the intended output is obtained, including male layer chicks	Yes	Yes	Yes	Farmed	Failed production or loss of productive value does not alter the purpose for which the animal was produced and controlled
Animal meat tissue, grown in labs	Yes	Yes	Yes	Not an animal	The cells do not (with current technology) create a living animal organism
Animals kept to provide cells for commercial lab grown meat	Yes	Yes	Yes	Farmed	The animal is grown or sustained to produce a material good for profit
Free-living animals, captured to extract their cells for lab grown meat, then released	No	Yes	Yes	Not farmed	The animals are not grown or sustained under human control
Dogs, rodents, or other animals commercially bred and held for sale as living animals	Yes	Yes	Yes	Farmed	The living animal is being produced and supplied as the material good
Captive-bred animals stocked in pet shops, transported, marketed, or held in live markets pending commercial sale	Yes	Yes	Yes	Farmed	They remain material goods, for-profit, during their time in the rest of the supply chain
A dog who has puppies which are sold, but companionship remains the main reason for keeping the dog	Yes	Yes	Not primary	Not farmed	A sold litter does not make the adult dog farmed unless profit becomes the dominant reason for keeping her
The same companion dog where income from breeding becomes a dominant reason for keeping and breeding her	Yes	Yes	Yes	Farmed	Classification changes when the primary purpose changes; profit need not be the sole motive
Puppies from a one-off litter produced and held primarily for profitable sale	Yes	Yes	Yes	Farmed	The mother and puppies may have different classifications: each animal is assessed separately
Laboratory rodents commercially bred and held for sale to research institutions	Yes	Yes	Yes	Farmed	Their living bodies are produced as material goods, even though their later use is scientific research
The same rodents after acquisition and while kept solely for scientific research	Yes	No	Profit may be primary	Not farmed	The primary output is knowledge rather than a material good
Laboratory rodents bred “in-house” by research facilities for their own use in science	Yes	No	Profit may be primary	Not farmed	The animals are never sold as a material good; they are bred directly for research, which produces knowledge
Racehorses bred, reared, and held primarily for profitable sale into racing	Yes	Yes	Yes	Farmed	The horses are being produced and supplied as living material goods during the breeding and sale phase
The same horses after purchase and while kept primarily to race	Yes	No	Profit may be primary	Not farmed	Racing is not a material good produced from or constituted by the horse
Oxen, horses, or other animals kept primarily to provide traction or other labour	Yes	No	Yes	Not farmed	The crops or goods enabled by labour are not material goods produced from or constituted by the animal
Fertile animals (e.g., ewes, tups), kept to reproduce offspring for future profit	Yes	Yes	Yes	Farmed	The animals produce material goods (eggs and sperm) which then lead to material goods
Animals, used during the breeding process, which don’t reproduce (e.g., teaser bulls)	Yes	No	Yes	Not farmed	The animals are primarily kept for a service (e.g., identifying fertile females) but provide no sperm or eggs themselves nor any offspring
Bumblebees commercially bred and held for sale as pollination colonies	Yes	Yes	Yes	Farmed	The living colonies are material goods during breeding, stocking, transport, and sale
Purchased bumblebees kept solely to pollinate the keeper’s crops	Yes	No	Yes	Not farmed	The animals provide a service rather than directly producing material goods or being material goods themselves
Animals kept primarily for guiding, detection, therapy, racing, display, entertainment, or companionship	Yes	No	Profit may be primary	Not farmed	Income generated through an animal’s performance or presence does not satisfy the material-good criterion
Animals bred and held for hunting	Yes	Yes	Yes	Farmed	The animal’s body itself is the object to be pursued, shot, and/or killed, even where no trophy is retained
Extensively grazed animals whose location, reproduction, behavioural opportunities, and welfare remain substantially determined by humans	Yes	Yes	Yes	Farmed	Continuous confinement is unnecessary where all dimensions of substantial human control are present
Free-living animals briefly captured, tagged, vaccinated, neutered, treated, or provisioned and then released	No	No	No	Not farmed	Brief, intervention-specific restraint does not make the animal’s location, behavioural opportunities, and welfare substantially human-determined during the broader relevant period
Previously farmed animals transferred to and kept in a sanctuary for their care	Yes	No	No	Not farmed	Farmed status ends when the production purpose ends, even though human control continues
Free-living animals captured alive and then reared, fattened, or bred under human management	Yes	Yes	Yes	Farmed after capture	A previously free-living animal can become farmed once all three criteria are satisfied
Earthworms farmed for the production of vermicompost	Yes	Yes	Yes	Farmed	The material good is a substance derived from the animals
Wild-caught fish or crustaceans held alive on board, in tanks, or in live markets prior to slaughter	No	Yes	Yes	Not farmed	The animals are kept alive but not grown or sustained. When they start being fed, fattened, or bred, they become farmed in line with the FAO’s definition [46]
Black soldier fly larvae reared on organic waste and harvested for animal feed or oil, or whose frass is sold as fertiliser	Yes	Yes	Yes	Farmed	The larvae, and the substances derived from them, are material goods
Insects reared solely to reduce or process organic waste, where neither the larvae nor their by-products are harvested	Yes	No	Yes	Not farmed	Waste processing is a service, rather than a material good
Bamboo worm (*Omphisa fuscidentalis*) larvae, introduced and then taken from bamboo culms, and consumed by humans	Yes	Yes	Yes	Farmed	The worms are a material good for profit or sustenance, and the question is around whether they are sustained or grown under human control. Here (i) their physical location (the culm) is determined by humans; (ii) their natural behaviours (what they eat and where they develop) are determined by humans; and (iii) their welfare is determined by humans.
Swiftlets, encouraged to nest in human made housing, so their edible nests can be harvested	No	Yes	Yes	Not farmed	As above, the question is about being sustained or grown under human control. Here (i) while their nest location is encouraged by humans, they are otherwise free; (ii) their natural behaviour is generally unaffected; (iii) their welfare is generally not affected by humans. Further they are not fed or grown by humans.
Traditional (fixed-comb) bee hives, where keepers encourage wild bees to form colonies in certain places	No	Yes	Yes	Not farmed	As above, the question is about being sustained or grown under human control. Here (i) while their nest location is encouraged by humans, they are otherwise free; (ii) their natural behaviour is generally unaffected; (iii) their welfare is generally not affected by humans (except at harvest). Further they are not fed or grown by humans.
Vicuñas, caught from the wild, shorn for their fur, and released	No	Yes	Yes	Not farmed	As above, the question is about human control. Here (i) their location is only briefly determined by humans; (ii) their natural behaviour is generally unaffected; (iii) their welfare is only affected by humans during shearing and potentially after.
Wild caught fish, whose spawning location has been affected by anthropogenic climate change	No	Yes	Yes	Not farmed	The question is again about human control. Here (i) humans are not directly seeking to restrict or direct their movement—instead it happens as an indirect consequence of other human actions; (ii) most natural behaviours are unchanged; (iii) human behaviour may have indirectly affected their location but does not determine their day-to-day welfare.
